# Lobular breast cancers lack the inverse relationship between ER/PR status and cell growth rate characteristic of ductal cancers in two independent patient cohorts: implications for tumor biology and adjuvant therapy

**DOI:** 10.1186/1471-2407-14-826

**Published:** 2014-11-10

**Authors:** Hilda Wong, Silvia Lau, Polly Cheung, Ting Ting Wong, Andrew Parker, Thomas Yau, Richard J Epstein

**Affiliations:** Division of Hematology/Oncology, University Department of Medicine, University of Hong Kong, Queen Mary Hospital, Pokfulam, Hong Kong; Medical Research Department, Hong Kong Sanatorium & Hospital, Hong Kong, China; Breast Center, Hong Kong Sanatorium & Hospital, Hong Kong, China; Departments of Pathology, Medical Oncology, UNSW Clinical School, St Vincent’s Hospital, The Kinghorn Cancer Center, Sydney, Australia; UNSW Clinical School, St Vincent’s Hospital, The Kinghorn Cancer Center, Sydney, Australia; The Kinghorn Cancer Center, Sydney, Australia

## Abstract

**Background:**

Although invasive lobular carcinoma (ILC) of the breast differs from invasive ductal carcinoma (IDC) in numerous respects - including its genetics, clinical phenotype, metastatic pattern, and chemosensitivity - most experts continue to manage ILC and IDC identically in the adjuvant setting. Here we address this discrepancy by comparing early-stage ILC and IDC in two breast cancer patient cohorts of differing nationality and ethnicity.

**Methods:**

The clinicopathologic features of 2029 consecutive breast cancer patients diagnosed in Hong Kong (HK) and Australia (AUS) were compared. Interrelationships between tumor histology and other clinicopathologic variables, including ER/PR and Ki67, were analysed.

**Results:**

Two hundred thirty-nine patients were identified with ILC (11.8%) and 1790 patients with IDC. AUS patients were older (*p* <0.001) and more often postmenopausal (*p* <0.03) than HK patients. As expected, ILC tumors were lower in grade and proliferative rate, and more often ER-positive and HER2-negative, than IDC (*p* <0.002); yet despite this, ILC tumors were as likely as IDC to present with nodal metastases (*p* >0.7). Moreover, whereas IDC tumors exhibited a strongly negative relationship between ER/PR and Ki67 status (*p* <0.0005), ILC tumors failed to demonstrate any such inverse relationship (*p* >0.6).

**Conclusion:**

These data imply that the primary adhesion defect in ILC underlies a secondary stromal-epithelial disconnect between hormonal signaling and tumor growth, suggesting in turn that this peritumoral feedback defect could reduce both the antimetastatic (adjuvant) and tumorilytic (palliative) efficacy of cytotoxic therapies for such tumors. Hence, we caution against assuming similar adjuvant chemotherapeutic survival benefits for ILC and IDC tumors with similar ER and Ki67, whether based on immunohistochemical or gene expression assays.

## Background

The advent of molecular genomics is ushering in a new paradigm of personalised cancer management in which treatments come to match biomarker-defined tumor subtypes [1]. A prime example of such a tumor subtype is invasive lobular carcinoma (ILC) of the breast - the second commonest histology after invasive ductal carcinoma (IDC) - which accounts for 5-15% of primary breast tumors and, unlike IDC, is rising in frequency [2]. Compared to IDCs, ILCs tend to be larger and lower grade [3]; less FDG-avid on PET scanning [4]; less often associated with vascular invasion [5], angiogenic growth factor expression or stromal reaction [3]; more often node-positive and metastatic [6], especially to bone or serosal surfaces [7]; and more resistant to chemotherapy [8] despite less frequent *TP53* gene mutations [9]. The signature of ILC on gene expression profiling also differs from that of grade-/subtype-matched IDC [10].

Sporadic ILCs are characterized by loss of cell adhesion mediated by the epithelial cadherin-catenin complex, as diagnostically confirmed by absent immunochemical detection of the transmembrane E-cadherin protein. This ILC adhesion defect is constitutive, often reflecting frameshift mutations of the *CDH1* gatekeeper tumor suppressor gene that cause truncation of the E-cadherin extracellular domain, together with loss of heterozygosity for the wild-type allele [11]. The accompanying defect in ILC adhesion gives rise to the typical histopathologic appearance of strand-like 'single-file' tumor cells and/or discohesive signet ring cells within a stroma lacking tissue reaction, a phenotype in turn attributable to reduced stromal-epithelial crosstalk by transforming growth factor-beta [10]. This lack of stromal reaction may underlie the lower palpability of ILC compared to IDC, contributing to the larger size of ILC tumors [12].

Given this convincing spectrum of clinicopathologic and molecular differences [13], it may seem surprising that current orthodoxies still support identical stage-specific adjuvant management of ILC and IDC [7, 14]. An increasing number of reports have highlighted that the apparently favorable ('luminal-like' [15]) phenotype of ILC tumors - namely, low nuclear grade, high ER-positivity, absent HER2, CCND1 and TOP2A amplification, and low growth rates [15, 16] - fails to translate into survival benefit relative to IDCs, whether stage-matched or not [17]. Other studies have suggested a similar overall prognosis in ILC and IDC [3, 12, 14], though this conclusion could misleadingly reflect (i) a superior stage-matched 5-year survival for ILC [18] balanced by a longer-term overall survival advantage for IDC due to less frequent late metastatic relapses [5], or (ii) a worse prognosis for node-positive ILC than IDC offset by a relatively better prognosis for node-negative ILC [19].

To resolve these discrepancies, at least some of which could reflect confounding by sample heterogeneity, the present study compares ILC tumor characteristics with those of IDC controls in two independent cohorts from countries with divergent epidemiology. Specifically, the natural history of breast cancer in Australia (AUS) mimics that of developed Western countries in Europe or North America, whereas the rising breast cancer incidence in younger Hong Kong (HK) Chinese patients reflects a recent lifestyle-dependent cohort effect [20, 21]. Here we exploit this dual-sample comparison to frame a systematic interrogation of the functional interrelationships between ILC and IDC tumor parameters.

## Methods

We analyzed cohorts of consecutive primary breast cancer patients treated at either the Hong Kong Sanatorium and Hospital in 2001–2011, or at St. Vincent’s Hospital, Sydney in 2007–2012. Patients with metastatic disease, or pathological subtypes other than ILC and IDC, were excluded. All patients were treated with curative intent, consisting of mastectomy or breast conservation, followed by external beam radiotherapy and/or systemic adjuvant therapy. Eligible patients were then classified according to geography and histology into ILC and IDC groups from HK (denoted by HK-ILC and HK-IDC, respectively) *vs*. those from AUS (AUS-ILC, AUS-IDC). Demographics, clinicopathological data including tumor size, grade, lymphovascular infiltration, lymph node involvement, ER, PR and HER2 status and Ki67 (cell proliferation) status, together with survival durations where available, were recorded. The access to the clinical databases used in this study was permitted by the ethics committee of both Queen Mary Hospital, Hong Kong and St Vincent’s Hospital, Sydney, Australia.

Tumor histology and the number of involved lymph nodes were evaluated by hematoxylin-eosin staining. Immunohistochemistry (IHC) was performed using commercial kits on formalin-fixed, paraffin-embedded specimens. In the HK tumor samples, IHC of ER and PR was assessed using 6 F11 and 1A6 antibodies respectively, and detected by the polymer EnVision system (Dako, Glostrup, Denmark). Expression of ER and PR were graded by the semi-quantitative H-score, where a score of over 50 out of 300 was interpreted as positive. In the AUS samples, the antibodies SP1 and 1E2 stained on Ventana Ultra platform (Ventana Medical Systems, Tucson, Arizona, USA) were used in IHC of ER and PR respectively. According to AUS criteria, positivity was defined as nuclei staining of 1% or more. HER2 IHC assays used in HK and AUS samples were A0485 (Dako) and 4B5 (Ventana) respectively. HER2 positivity was defined by IHC 3+ (strong positive staining on at least 10% of breast tissue specimen) and/or fluorescent in situ hybridization (FISH)-amplified (HER2 DNA to chromosome 17 centromere DNA ratio of at least 2.2), the latter using using PathVysion Vysis FISH (Abbott, Chicago, IL, USA). Both IDC and ILC tumors were graded using modified Bloom & Richardson scoring criteria, viz., summation of scores (1–3) for nuclear morphology, tubule formation, and mitotic score; the latter parameter correlates best with both Ki67 score and disease prognosis [22, 23]. Expression of Ki67 was assessed in Hong Kong tumor samples using the antibody SP6 (Neomarkers/LabVision), a rabbit monoclonal antibody, which provides similar accuracy, reproducibility and prognostic value when compared to MIB1 in primary breast cancer [24, 25]. For the Sydney series we used the 30–9 (Ventana, Roche group) antibody which is another FDA-approved rabbit monoclonal IgG directed against the C-terminal portion of the Ki67 protein, leading to selective immunostaining of non-resting, i.e., non-G_0_, cells (http://www.ventanamed.com). For both patient sample cohorts, 5–10 high power fields were examined at the periphery of each tumor; the percentage of nuclei staining was quantified in both series using manual Ki67 scoring of whole sections from excision specimens (and not from digital image analysis) according to the guidelines published by Dowsett M et al. [26]. In both cohorts, tumor samples were arbitrarily categorised by Ki67 levels into separate high (>10%) and low (<5%) groups to facilitate clear qualitative comparison. E-cadherin immunostaining was routinely used as one of the key parameters, though not the only such parameter, distinguishing ILC from IDC morphologic diagnoses.

Summary statistics were used to quantify patient demographics. The chi-square and Mann–Whitney-U tests were performed to assess the relationship between ordinal and numerical variables, respectively. Demographics and clinicopathological characteristics of the HK-ILC and AUS-ILC groups were compared; these groups were also contrasted with the respective IDC cohorts from the same geographical location. We used bivariate analysis – a specific subtype of multivariate analysis which, unlike univariate analysis, is not simply descriptive – to test the causal relationship between two clinicopathologic variables - Ki67 and ER/PR status - pertinent to the distinct disease biologies of ILC and IDC (see Discussion). To aid clinical decision-making, we streamlined this bivariate analysis by partitioning the latter continuous variables into non-parametric positive/negative (ER/PR) vs. high/low (Ki-67), permitting a Pearson's chi-square computation. Moreover, to minimise the risk of identifying a chance retrospective statistical association, all calculations on the total cohort were repeated in the two (HK and AUS) independent sub-cohorts. Calculations were performed using the statistical software SPSS, version 18, and significance inferred at *p* <0.05.

## Results

A total of 2029 patients was analyzed. The number of patients in the HK-ILC, HK-IDC, AUS-ILC and AUS-IDC groups were 141, 1159, 98 and 631 respectively. All were female. As shown in Table 1, the median age at presentation of the AUS-ILC patients was 64, compared to 50 for HK-ILC patients (p <0.0005); as expected, more AUS-ILC patients were post-menopausal (p =0.029). The size of the primary tumor (median 2.4 cm and 2.5 cm respectively for AUS-ILC and HK-ILC groups, p =0.825) and the proportion of patients with regional lymph node involvement (47.1% and 40.0% respectively, p =0.299) were similar in both cohorts. As in earlier studies, ILC tumors tended to be ER-positive, PR-positive and HER2-negative; although these expression patterns were not significantly different between the AUS-ILC and HK-ILC groups, a trend towards more frequent ER- and PR-negativity was evident in the younger HK cohort (*p* <0.09). In contrast, HER2 positivity was equally uncommon in both ILC cohorts (5.4 *vs*. 6.6%, *p* =0.71); this was also the case for median Ki67 levels (5% *vs*. 6% in AUS-ILC and HK-ILC patients, respectively; *p* =0.746), with the proportions of patients with high (≥10%) and low (≤5%) Ki67 similar (*p* =0.293).

**Table 1:** **Comparison of patient demographics and tumor characteristics of AUS-ILC and HK-ILC cohorts**

		No. of patients (%)			
Characteristics		All ILC	AUS-ILC	HK-ILC	***p***
Age at diagnosis					
	**Median (range)**	55 (34 – 86)	64 (34 – 86)	50 (34 – 82)	<0.0005
	**<= 35**	2 (0.9%)	1 (1.1%)	1 (0.7%)	
	**36 - 50**	84 (36.2%)	14 (14.9%)	70 (49.6%)	
	**≥ 51**	146 (62.9%)	79 (84.0%)	67 (48.6%)	
**Menopausal status**					
	**Pre-menopausal**	39 (30.5%)	8 (18.2%)	31 (36.9%)	0.029
	**Post-menopausal**	89 (69.5%)	36 (81.8%)	53 (63.1%)	
**Tumor size (cm)**					
	**Median (range)**	2.4 (0.18 – 20.0)	2.4 (0.5 – 20.0)	2.5 (0.18 – 10.1)	0.825
**LN involvement**					
	**Negative**	129 (57.3%)	45 (52.9%)	84 (60.0%)	0.299
	**Positive**	96 (42.7%)	40 (47.1%)	56 (40.0%)	
**ER**					
	**Negative**	12 (5.2%)	2 (2.2%)	10 (7.2%)	0.089
	**Positive**	220 (94.8%)	91 (97.8%)	129 (92.8%)	
**PR**					
	**Negative**	48 (20.9%)	14 (15.2%)	34 (24.6%)	0.085
	**Positive**	182 (79.1%)	78 (84.8%)	104 (75.4%)	
**HER2**					
	**Negative**	191 (94.1%)	85 (93.4%)	106 (94.6%)	0.71
	**Positive**	12 (5.9%)	6 (6.6%)	6 (5.4%)	
**Ki67 (%)**					
	**Median**	5.0	5.0	6.0	0.746
	**≤ 5**	83 (50.6%)	17 (54.8%)	66 (49.6%)	
	**6 - 9**	32 (19.5%)	0 (0.0%)	32 (24.1%)	
	**≥ 10**	49 (29.9%)	14 (45.2%)	35 (26.3%)	

### Comparison of ILC with IDC controls

As shown in Table 2, patients with ILC were more frequently postmenopausal than those with IDC in both the HK and AUS cohorts (*p* ≤0.003). Primary ILC tumors were both larger and of lower grade than IDC in both patient cohorts (all *p* <0.0005), but there was no ILC/IDC difference in the proportion of patients with lymph node metastases (*p* >0.7). ILC tumors in both cohorts were more often ER-positive (*p* ≤0.001), HER2-negative (*p* <0.02) and low-Ki67 (*p* ≤0.002) than the corresponding IDC tumors. While a trend towards more frequent PR-positivity for ILC than IDC tumors was noted in the older AUS cohort (84.8 vs. 76.6%; *p* <0.08), no such trend was demonstrable for HK-ILC over HK-IDC (75.4 vs. 72.7%, *p* >0.5).

**Table 2:** **Contrast of patient demographics and tumor characteristics of ILC against IDC, as stratified by geographical location**

Characteristics		All ILC	All IDC	Sp. Cor.	***p***	AUS-ILC	AUS-IDC	Sp. Cor.	***p***	HK-ILC	HK-IDC	Sp. Cor.	***p***
Menopausal status (pre-, post-menopausal, MP)				−0.155	<0.0005			−0.160	0.003			−0.135	<0.0005
	No. (%) Post-MP	89 (69.5%)	601 (41.3%)			36 (81.8%)	174 (58.6%)			53 (63.1%)	427 (36.8%)		
Tumor size (≤2, >2-5, >5 cm)				−0.148	<0.0005			−0.145	<0.0005			−0.150	<0.0005
	Median (cm)	2.4	1.8			2.4	1.8			2.5	1.8		
Tumor grade (1,2,3)				0.205	<0.0005			0.138	<0.0005			0.239	<0.0005
	No. (%) Grade 3	14 (6.5%)	873 (49.5%)			8 (8.5%)	275 (44.3%)			6 (4.9%)	598 (52.3%)		
LN involvement (no, yes)				−0.007	0.761			0.007	0.852			−0.011	0.706
	No. (%) Positive	96 (42.7%)	711 (41.6%)			40 (47.1%)	273 (48.1%)			56 (40.0%)	438 (38.4%)		
ER (negative, positive)				−0.121	<0.0005			−0.131	0.001			−0.114	<0.0005
	No. (%) Positive	220 (94.8%)	1416 (80.3%)			91 (97.8%)	512 (84.6%)			129 (92.8%)	904 (78.0%)		
PR (negative, positive)				−0.037	0.096			−0.067	0.079			−0.018	0.511
	No. (%) Positive	182 (79.1%)	1301 (74.0%)			78 (84.8%)	458 (76.6%)			104 (75.4%)	843 (72.7%)		
HER2 (negative, positive)				0.095	<0.0005			0.091	0.018			0.093	0.001
	No. (%) Negative	210 (91.7%)	1383 (80.3%)			85 (93.4%)	482 (84.0%)			125 (90.6%)	901 (78.5%)		
Ki67 (≤5, 6–9, ≥10)				0.269	<0.0005			0.191	0.002			0.252	<0.0005
	Median	5	15			5	15			6	15		

### Relationship between Ki67 and clinicopathological features in ILC and IDC

An analysis of tumor parameters in terms of proliferation rate, as defined by Ki67 high (≥10%) and low (≤5%) cutoffs, is shown for ILC and IDC in Tables 3 and 4 respectively. A direct correlation between Ki67 and either tumor size, lymph node metastasis, or HER2 status was evident in both ILC and IDC cohorts when combined. This relationship did not reach statistical significance for the individual AUS-ILC (*p* <0.06) or HK-ILC cohorts (*p* <0.09) with respect to tumor size, perhaps reflecting lower numbers relative to IDC counterparts, nor for the AUS-ILC cohort with respect to HER2 status (*p* =0.28); however, the latter value reduced to *p* =0.06 following age correction, suggesting confounding due to very low numbers (one case only) of HER2-positive ILC in the older AUS cohort. In contrast to the above-mentioned similar Ki67 correlations in ILC and IDC, there was a highly significant inverse relationship between ER/PR status and high-Ki67 subset for IDC in both cohorts (*p* ≤0.002; Table 4), but no significant relationship between ER or PR status and high/low Ki67 subset for ILC irrespective of whether evaluated separately or together (*p* >0.6; Table 3).

**Table 3:** **Correlation of clinicopathological charateristics in ILC patients with Ki67 ≤ 5%** ***vs*** **. ≥10%**

		All ILC			AUS-ILC			HK-ILC		
Characteristics		No. with Ki67 ≤ 5 (%)	No. with Ki67 ≥ 10 (%)	***p***	No. with Ki67 ≤ 5 (%)	No. with Ki67 ≥ 10 (%)	***p***	No. with Ki67 ≤ 5 (%)	No. with Ki67 ≥ 10 (%)	***p***
Tumor size										
	Median (range)	2.2 (0.18 – 12.1)	3.0 (0.20 – 11.0)	0.012	2.2 (0.5 - 12.0)	4.5 (1.5 - 11.0)	0.057	2.2 (0.18-7.0)	3.0 (0.2-9.5)	0.089
LN involvement										
	Negative	58 (70.7%)	15 (33.3%)	<0.0005	13 (81.3%)	0 (0.0%)	<0.0005	45 (68.2%)	15 (42.9%)	0.014
	Positive	24 (29.3%)	30 (66.7%)		3 (18.8%)	10 (100.0%)		21 (31.8%)	20 (57.1%)	
ER										
	Negative	5 (6.1%)	4 (8.2%)	0.654	0 (0%)	1 (7.1%)	0.277	5 (7.6%)	3 (8.6%)	0.86
	Positive	77 (93.9%)	45 (91.8%)		16 (100%)	13 (92.9%)		61 (92.4%)	32 (91.4%)	
PR										
	Negative	21 (25.6%)	11 (22.4%)	0.686	4 (25%)	1 (7.1%)	0.19	17 (25.8%)	10 (28.6%)	0.761
	Positive	61 (74.4%)	38 (77.6%)		12 (75%)	13 (92.9%)		49 (74.2%)	25 (71.4%)	
HER2										
	Negative	77 (96.3%)	42 (85.7%)	0.030	16 (100%)	13 (92.9%)	0.277	61 (95.3%)	29 (82.9%)	0.039
	Positive	3 (3.8%)	7 (14.3%)		0 (0.0%)	1 (7.1%)		3 (4.7%)	6 (17.1%)

**Table 4:** **Correlation of clinicopathological charateristics in IDC patients with Ki67 ≤ 5%** ***vs.*** **≥10%**

		All IDC			AUS-IDC			HK-IDC		
Characteristics		No. with Ki67 ≤ 5 (%)	No. with Ki67 ≥ 10 (%)	***p***	No. with Ki67 ≤ 5 (%)	No. with Ki67 ≥ 10 (%)	***p***	No. with Ki67 ≤ 5 (%)	No. with Ki67 ≥ 10 (%)	***p***
**Tumor size**										
	**Median (range)**	1.4 (0.01 – 10.0)	2.0 (0.01 – 14.5)	<0.0005	1.5 (0.2 - 6.5)	2.2 (0.2 - 14.5)	<0.0005	1.3 (0.01 - 10.0)	1.9 (0.01 - 10.0)	<0.0005
**LN involvement**										
	**Negative**	177 (69.1%)	493 (56.6%)	<0.0005	33 (66%)	66 (46.5%)	0.018	144 (69.9%)	427 (58.6%)	0.003
	**Positive**	79 (30.9%)	378 (43.4%)		17 (34%)	76 (53.5%)		62 (30.1%)	302 (41.4%)	
**ER**										
	**Negative**	13 (4.9%)	270 (30.0%)	<0.0005	1 (1.8%)	35 (21.6%)	0.001	12 (5.7%)	235 (31.8%)	<0.0005
	**Positive**	252 (95.1%)	630 (70.0%)		54 (98.2%)	127 (78.4%)		198 (94.3%)	503 (68.2%)	
**PR**										
	**Negative**	27 (10.2%)	313 (34.9%)	<0.0005	3 (5.4%)	40 (25.0%)	0.002	24 (11.4%)	273 (37.0%)	<0.0005
	**Positive**	239 (89.8%)	585 (65.1%)		53 (94.6%)	120 (75.0%)		186 (88.6%)	465 (63.0%)	
**HER2**										
	**Negative**	243 (92.0%)	650 (73.0%)	<0.0005	54 (98.2%)	125 (78.1%)	0.001	189 (90.4%)	525 (71.8%)	<0.0005
	**Positive**	21 (8.0%)	241 (27.0%)		1 (1.8%)	35 (21.9%)		20 (9.6%)	206 (28.2%)	

## Discussion

The central insight from this international dual-cohort comparison of ILC and IDC tumor parameters is that the strongly inverse relationship long noted between ER/PR and Ki67 immunohistochemistry in IDC [27] appears weaker or absent in ILC. Regarded by many as the most critical single molecular prognosticator in breast cancer, even when compared with costlier multigene expression profiling [28], the Ki67 proliferative index is at once a negative correlate of disease-free survival and overall survival [29, 30] and a strong predictor of initial response to chemotherapy - although these inferences can only be applied to IDC at present.

Some retrospective studies have reported improved survival of ILC patients relative to IDC patients, concluding that ILC responds better to adjuvant hormone therapy [31], though such non-randomised observations are weakened by the possibility that ILC patients may be at lower overall risk than IDC patients. Consistent with this possibility, it is now recognised that breast cancers such as IDC and ILC evolve via multiple pathways involving different combinations of molecular variables such as *TP53* gene mutations (commoner in IDC than ILC; see above) and/or mTOR pathway activation (commoner in ILC than IDC; see below).

Recent molecular ER technologies have clarified the differential isoform (ER-α and -β) contributions to overall breast tumour ER-positivity. Whereas ER-α drives proliferation of mammary epithelial cells, implying a valid therapeutic target, ER-β is associated with differentiation of normal breast cells [32], mediates the preventive benefits of exercise and parity [33] on breast cancer incidence, and may directly inhibit breast cancer progression [34]. Unlike IDCs, however, in which both ER-α and -β tend to be similarly co-expressed, ILCs display a reciprocal relationship between ER-α and ER-β, with abnormally high ER-α levels but subnormal expression of ER-β [35]. The pro-differentiation action of ER-β is mediated in part via direct transcriptional upregulation of E-cadherin, in turn repressing the oncogenic Wnt pathway via nuclear β-catenin [36]; the association of low ER-β levels with tamoxifen resistance and reduced survival benefit from adjuvant hormone therapy [37] may therefore be clinically relevant to ILC. Unlike in ILC where the function of the cadherin-catenin complex is irreversibly repressed (i.e., even if E-cadherin remains expressed [38]) and hence inhibits apoptosis [39], tamoxifen therapy of ER-positive IDC cells appears capable of restoring E-cadherin-dependent adhesion and augment apoptosis [40].

E-cadherin downregulation is not specific to ILC, as it also occurs during progression to high-Ki67 IDC tumors such as basaloid and triple-negative subtypes, reflecting dynamic epigenetic trans-repression of *CDH1* at the invasive tumor front as part of epithelial-mesenchymal transition (EMT) [41]. Estradiol stimulates the latter pro-invasive process in ductal breast cancer cells via upregulation of TGF-β signaling and expression of EMT-related transcription factors such as Snail [42], leading to activation of Wnt signaling. Clinically, Snail levels correlate with metastatic aggressivity and poor prognosis in IDC [43]. However, Snail expression is not elevated in ILC [44], reflecting the fact that Snail expression is mainly restricted to E-cadherin-expressing carcinoma cells [45]. The lack of EMT so implied in ILC is therefore consistent with the inability of these irreversibly cadherin-defective tumors to excite stromal reaction or to present with a scirrhous phenotype [46].

How is the observed adhesion-dependent link between ER/PR expression levels and breast cancer cell growth to be explained at a molecular level? From a broad perspective, breast cancer may be subclassified into EMT-associated ER-poor tumors with *TP53* dysfunction at one extreme, contrasting with *TP53*-wildtype ER-rich tumors with predisposing primary defects of the PI3K-Akt-mTOR anti-apoptotic pathway at the other [9]. By including histology (IDC *vs*. ILC) as a subgroup variable, however, we can further subclassify ER-positive tumors. The ER + IDC pathway tends to be activated by early mutations affecting the anti-apoptotic (pro-survival) PI3K signaling pathway; the commonest such mutation affects the *PTEN* gatekeeper gene, permitting secondary ER-α and ER-β upregulation, leading in turn to Snail induction, EMT-related TGF-β and Wnt pathway activation, BRCA1/2 and/or *TP53* inactivation. Snail overexpression within E-cadherin-expressing carcinoma cells directly mediates ER-α repression [47]; hence, the resulting EMT leads to simultaneous ER/PR decline and Ki67 elevation [48], with or without HER-family growth factor receptor upregulation. When the EMT transactivator Twist is co-expressed with Snail, TGF-β-dependent E-cadherin downregulation supervenes [43], with low E-cadherin and high Ki67 marking an especially poor-prognostic breast cancer subgroup [49]. ER and Ki67 tend not to be co-expressed in normal breast cells, with such co-expression only becoming detectable during early-stage tumorigenesis and accelerating during progression [50].

Consistent with this, others have noted that primary IDC cell proliferation is maximal at the advancing tumor edge [51], a finding that we have recently confirmed to be relevant to IDC but not to ILC (AP, unpublished observations). As noted above, the defining adhesion defect of ILC selectively impairs apoptosis/anoikis while simultaneously selecting for both ER-α overexpression and PI3K pathway upregulation via secondary mechanisms such as increased PTEN proteolysis or activating PIK3CA mutations [52]. The primary loss of E-cadherin functionality in ILC has additional consequences that distinguish its behavior from that of ER + (or 'luminal') IDC, including failure of Snail upregulation and hence prevention of EMT-associated ER repression as noted above. ILC-linked destabilization of β-catenin also prevents upregulation of Wnt signalling, thus accounting for the ILC-associated lack of Ki67 increase relative to IDC. This is consistent with work showing that loss of the Wnt5a tumor suppressor protein is associated with shortened survival and ER/PR-negativity in IDC but not in ILC [53], supporting a stronger role for Wnt activation, EMT, and ER/PR loss in IDC than in ILC.

**Figure 1:**
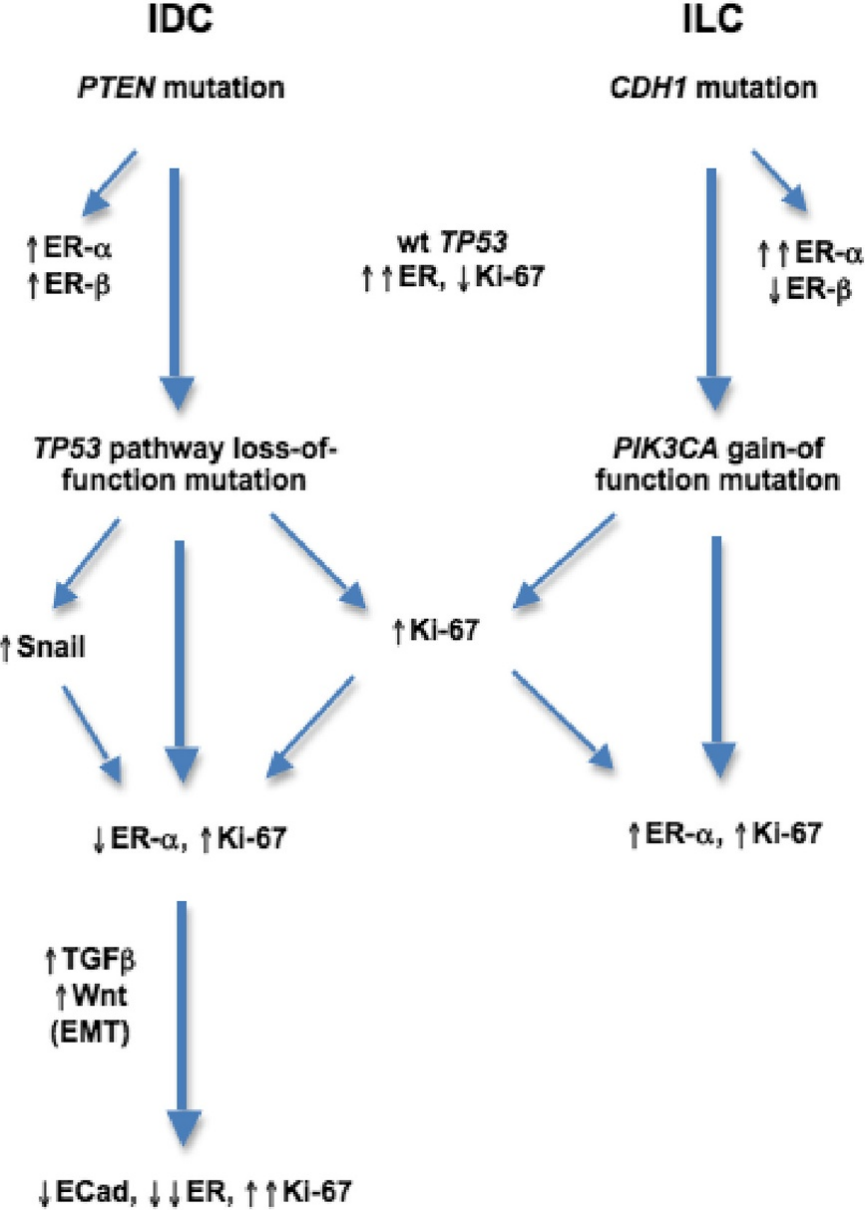
**Model of how the differing molecular evolution of IDC and ILC could explain the loss of negative feedback between ER and Ki67 status.** See text for details.

Although at first glance the observations above might seem relevant only to hormonal resistance, the biology of ILC could be equally relevant to chemotherapy resistance; indeed, as mentioned earlier, there is even stronger clinical evidence for the latter. Increasing evidence [54] supports the view that both the adjuvant and palliative benefits of cytotoxic therapy derive at least in part from cell damage caused to the peritumoral stromal cells which provide paracrine growth networks that minimise tumor cell apoptosis. Since these paracrine loops would seem likely to be less potent in ILC than in IDC, however, it is very plausible that the benefits of adjuvant chemotherapy are also generally lower in ILC. A model illustrating how the defining adhesion defect of ILC could to underlie a breakdown in negative feedback between ER status and tumour proliferative rate is presented in Figure 1.

Unlike other retrospective studies in which statistical associations may arise due to selection bias or chance, the inverse correlation scrutinized here was independently replicated in two unrelated IDC cohorts, but not in either or both of the ILC cohorts combined. Accordingly, we submit that the utility of the present results is not limited to mere hypothesis generation, as is typically a major weakness of retrospective analyses. Nonetheless, there remain several important limitations to the interpretation of our study. First, the number of ILC patients was substantially lower than that of IDC patients, raising the possibility of a type I statistical error. Second, the histologic subset of ILC is itself heterogeneous, being divisible into additional non-classic ILC variants such as solid, alveolar, and pleomorphic which are associated with higher Ki67 status and poorer prognosis; given the relatively small size of this study, we cannot exclude that our conclusions may be only applicable to the classical ILC subgroup. A valuable focus for future research will thus be to clarify whether non-classical ILC tumors more closely resemble high-grade IDCs in their clinical behavior and therapeutic benefit.

Third, although the differences in age and ethnicity between the AUS and HK cohorts permit some degree of qualitative corroboration, they also raise questions about the significance of any quantitative differences observed between the groups; for example, are the study conclusions more readily applicable to younger and/or premenopausal (HK) than to older and/or postmenopausal (AUS) ILC patients, given the statistics in Table 3, and if so, should ILC arising in older patients predisposed by hormone replacement therapy [55] be reasonably assumed to be more hormone-responsive than ILC arising in younger patients? While this certainly seems plausible, further work is needed to reach a firm conclusion on this point.

Fourth, the present study compared two arbitrarily-defined but discontinuous Ki67 groupings of ≤5% (“slow”) *vs*. ≥10% (“fast”). In contrast, recent literature has generated a consensus figure of Ki67 = 14% as a qualitative numerical cut-off point to distinguish “faster” from “slower” breast tumors as part of a continuous distribution [56–58]. At the time that our study was originally designed, this cut-off convention had not been widely adopted. Moreover, we would argue that there is an arbitrary dimension to all such cut-offs – consider, for example, that a 13% Ki67 tumor’s biology is likely to differ more from a 4% Ki67 tumor than from a 15% Ki67 tumor, irrespective of which cutoff convention is used for study purposes. Accordingly, we maintain that our qualitative conclusions relating to “faster” and “slower” tumors are at least as valid, if not moreso, using the Ki67 cutoffs specified in the manuscript, given that this splits the comparison into two unequivocally distinct (i.e., numerically discontinuous) groups.

Finally, as with any non-centralized multicenter study, the differences in pathology reagents and techniques used in the two centers (see Methods) could in theory predispose to an inadvertent bias of the results and conclusions. For example, differences in the two Ki67 antibodies could in theory have led to significant discordances in results between the two series. In practice, however - given the demonstrated concordance of results based on two separately derived data sets - we submit that the dual-cohort design strengthens rather than weakens the reliability of the two substudies’ independent yet similar conclusions.

## Conclusions

In summary, the present study suggests that subtle but important functional differences are likely to distinguish the clinical behavior and therapeutic responsiveness of ILCs and IDCs. Whereas a rise in the Ki67 proliferation index is typically linked to a drop in ER/PR expression in IDC, cautioning against overreliance on hormonal therapies, our work indicates that this molecular caveat seldom occurs in ILC. Recent advances in understanding of the events involved in ILC progression, and their distinction from the EMT/Wnt cascades occurring in IDC, raise the hypothesis that mTOR inhibitors could prove effective in restoring hormone- and/or chemosensitivity to refractory advanced ILC tumors, as well as plausibly improving adjuvant survival outcomes for higher-risk ILCs being treated with these drug classes. We further recommend specific interrogation of meta-analysis databases used for randomized trials (e.g., EBCTCG) to quantify the relative value-add of hormonal and cytotoxic therapies in the adjuvant and palliative management of ILC *vs*. IDC.

## Abbreviations

AUS: Australia
ER: Estrogen receptor
HK: Hong Kong
FDG: Fluorodeoxyglucose
IDC: Infiltrating ductal carcinoma
IHC: Immunohistochemistry
ILC: Infiltrating lobular carcinoma
PET: Positron emission tomography
PR: Progesterone receptor.
